# Explicit Image Caption Reasoning: Generating Accurate and Informative Captions for Complex Scenes with LMM

**DOI:** 10.3390/s24123820

**Published:** 2024-06-13

**Authors:** Mingzhang Cui, Caihong Li, Yi Yang

**Affiliations:** 1 School of Information Science and Engineering, Lanzhou University, Lanzhou 730000, China; cuimzh21@lzu.edu.cn (M.C.); yy@lzu.edu.cn (Y.Y.); 2 Key Laboratory of Artificial Intelligence and Computing Power Technology, Lanzhou 730000, China

**Keywords:** image caption, explicit image caption, prompt engineering, large multimodal model

## Abstract

The rapid advancement of sensor technologies and deep learning has significantly advanced the field of image captioning, especially for complex scenes. Traditional image captioning methods are often unable to handle the intricacies and detailed relationships within complex scenes. To overcome these limitations, this paper introduces Explicit Image Caption Reasoning (ECR), a novel approach that generates accurate and informative captions for complex scenes captured by advanced sensors. ECR employs an enhanced inference chain to analyze sensor-derived images, examining object relationships and interactions to achieve deeper semantic understanding. We implement ECR using the optimized ICICD dataset, a subset of the sensor-oriented Flickr30K-EE dataset containing comprehensive inference chain information. This dataset enhances training efficiency and caption quality by leveraging rich sensor data. We create the Explicit Image Caption Reasoning Multimodal Model (ECRMM) by fine-tuning TinyLLaVA with the ICICD dataset. Experiments demonstrate ECR’s effectiveness and robustness in processing sensor data, outperforming traditional methods.

## 1. Introduction

The rapid development of advanced sensing technologies and deep learning has led to the emergence of image captioning as a research hotspot at the intersection of computer vision, natural language processing, and sensor data analysis. Image captioning enables computers to understand and describe the content of images captured by sophisticated sensors, combining computer vision and natural language processing techniques to address the challenge of transforming visual features into high-level semantic information. This technology is of significant consequence in a multitude of application contexts, including automated media management, assisting the visually impaired, improving the efficiency of search engines, and enhancing the interaction experience in robotics [1,2,3,4]. The advancement of image description technology not only advances the field of computer vision but also significantly enhances the practical applications of human–computer interactions in the real world.

Over the past few years, significant progress has been made in this area, with the adoption of encoder–decoder frameworks such as CNN–RNN [5,6] or Transformer [7,8]. These advances have enabled image captioning models to generate “high quality” captions from scratch. Furthermore, emerging research proposes Image Caption Editing tasks [9], especially Explicit Image Caption Editing [10], which not only corrects errors in existing captions but also increases the detail richness and accuracy of captions. Although these methods perform well in simplified tasks, they still face challenges in how to effectively improve the accuracy and information richness of generated captions when dealing with complex scenes and fine-grained information.

In light of the aforementioned challenges, this paper proposes a novel approach, Explicit Image Caption Reasoning (ECR). The method employs an enhanced inference chain to perform an in-depth analysis of an image, resulting in more accurate and detailed descriptions. The ECR method not only focuses on the basic attributes of the objects in an image but also systematically analyzes the relationships and interactions between the objects, thereby achieving a deeper level of semantic understanding. The introduction of the inference chain technique enables the reconstruction of the image description generation process. This process is capable of identifying key objects and their attributes in an image, as well as analyzing the dynamic relationships between these objects, including interactions and spatial locations. Finally, this information is combined to generate descriptive and logically coherent image captions. In comparison to traditional methods, ECR provides a more detailed and accurate image understanding and generates text that is closer to human observation and description habits.

To implement this approach, we utilize the optimized dataset ICICD, which is based on the original Flickr30K-EE dataset [10]. The Flickr30K-EE dataset (accessed on 15 February 2024) is accessible for download at https://github.com/baaaad/ECE.git. Although the ICICD dataset represents only 3% of the ECE instances in the original dataset, each instance is meticulously designed to contain comprehensive inference chain information. This high-quality data processing markedly enhances the efficiency of model training and the quality of captions, despite a considerable reduction in data volume.

Based on these considerations, we conduct experiments using the large multimodal model TinyLLaVA [11]. This model is designed to take full advantage of miniaturization and high efficiency, making it suitable for resource-rich research environments as well as computationally resource-constrained application scenarios. The model demonstrates excellent performance in processing large amounts of linguistic and visual data. The ICICD dataset is utilized to meticulously refine the TinyLLaVA model, resulting in a bespoke model, the Explicit Image Caption Reasoning Multimodal Model (ECRMM). Concurrently, a bespoke prompt is employed to facilitate visual comprehension within the large multimodal model Qwen-VL [12], which generates object relationship data for the inference chain. The combination of these measures ensures the efficient and high-quality performance of the new model, ECRMM, in image description generation.

**Figure 1:**
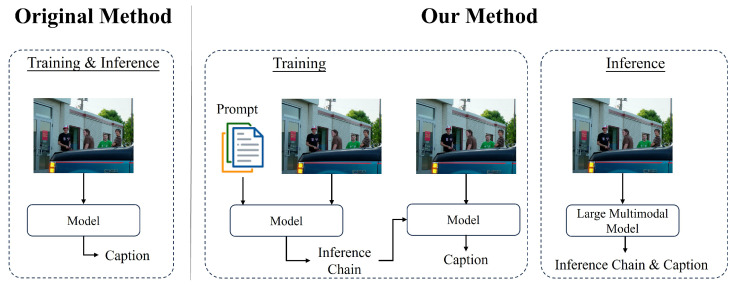
Compared to the original method, our model generates captions with the addition of inference chain.

In this study, we conduct a series of analysis experiments and ablation studies to verify the effectiveness and robustness of our method. The experimental results demonstrate that the inference chain-based inference method proposed in this paper is more accurate than traditional methods based on simple editing operations (e.g., ADD, DELETE, KEEP) in capturing and characterizing the details and complex relationships in an image. For instance, as illustrated in Figure 1, our model generates the caption “four men stand outside of a building”, whereas the model without the ECR method generates “four men stand outside”. While our model generates “building”, it also considers the relationship between people and buildings in the image in a more profound manner. This inference chain approach not only focuses on the information of various objects in the image but also on the positional relationship between each object. This significantly improves the quality of captions.

The main contributions of this paper include the following:

- We introduce a novel approach, designated as Explicit Image Caption Reasoning, which employs a comprehensive array of inference chaining techniques to meticulously analyze the intricate relationships and dynamic interactions between objects within images.
- We develop an innovative data generation method that employs large multimodal visual models to guide the generation of data containing complex object relationships based on specific prompts. Furthermore, we process the ICICD dataset, a detailed inference chain dataset, using the data of object relations.
- We fine-tune the TinyLLaVA model to create the ECRMM model and demonstrate the efficiency and superior performance of a large multimodal model for learning new formats of data.
- We demonstrate the effectiveness and robustness of explicit image caption inference through a series of analytical experiments based on inference chaining techniques. Our evaluation using a fine-tuned ECRMM model on a test dataset not only improves the scores but also shows the significant advantages and improvements of our approach over traditional methods through a careful ablation study.

The rest of this survey is organized as follows. First, we provide a systematical and detailed survey of works in the relevant fields in Section 2. Then, we introduce the dataset, modeling methodology, and the specific methods of ECR in Section 3 and Section 4. Next, we perform the evaluation experiments, ablation experiments, and a series of analytical experiments in Section 5. Finally, we summarize the work of our study in Section 6. Through this comprehensive and detailed discussion, we hope to provide valuable references and inspirations for the field of image caption generation. The code and dataset for this study are accessible for download at https://github.com/ovvo20/ECR.git (accessed on 15 February 2024).

## 2. Related Work

### 2.1. Sensor-Based Image Captioning

The early encoder–decoder model had a profound impact on the field of sensor-based image captioning, with groundbreaking work on the encoder focusing on target detection and keyword extraction from sensor data [13,14]. Developments in the decoder include the hierarchization of the decoding process, convolutional network decoding, and the introduction of external knowledge [15,16,17]. The field of sensor-based image captioning further advances with the introduction of attention mechanisms, which are continuously refined to focus on specific regions in sensor-derived images and incorporate dual attention to semantic and image features [18,19,20,21]. Generative Adversarial Networks (GANs) have been widely employed in sensor-based image captioning in recent years, enabling the generation of high-quality captions by learning features from unlabeled sensor data through dynamic game learning [22,23,24,25]. Additionally, the reinforcement learning approach yields considerable outcomes in the sensor-based image captioning domain, optimizing caption quality at the sequence level [26,27,28,29]. Dense captioning methods, which decompose sensor-derived images into multiple regions for description, have also been explored to generate more dense and informative captions [3,30,31,32].

### 2.2. Multimodal Models for Sensor Data Processing

The continuous development of large multimodal models has undergone a progression from initial attempts to recent optimizations for processing sensor data. Researchers have introduced autoregressive large language models into visual–linguistic learning for sensor-derived data, such as the Flamingo [33] model, which extracts visual features by inserting adapters into large language models and utilizing a Perceiver-like architecture. The BLIP-2 [34] model proposes a framework that optimizes the utilization of resources for processing sensor data, employing a lightweight Q-Former to connect the disparate modalities. LLaVA [35] and InstructBLIP [36] are fine-tuned by adjusting the data with visual commands, making them suitable for sensor-based applications. MiniGPT-4 [37] trains a single linear layer to align the pre-trained visual encoder with the LLM, demonstrating capabilities comparable to those of GPT-4 [38] in processing sensor-derived data. QWen-VL [12] allows for multiple image inputs during the training phase, which improves its ability to understand visual context from sensors. Small-scale large multimodal models, such as Phi-2 [39] and TinyLlama [40], have been developed to address the issue of high computational cost when deploying large models for sensor data processing, maintaining good performance while keeping a reasonable computational budget. These small models, such as TinyGPT-V [41] and TinyLLaVA [11], demonstrate excellent performance and application potential in resource-constrained environments involving sensor data analysis.

### 2.3. Text Editing and Image Caption Editing for Sensor Data

Text editing techniques, such as text simplification and grammar modification, have been applied to sensor-based image captioning to improve the quality of generated captions. Early approaches to text simplification for sensor data included statistical-based machine translation (SBMT) methods, which involved the deletion of words and phrases [42,43], as well as more complex operations such as splitting, reordering, and lexical substitution [44,45,46,47]. Neural network-based approaches, including recurrent neural networks (RNN) and transformers, have also been employed in text simplification tasks for sensor-derived data [48,49,50]. Various methods have been developed for grammar modification in the context of sensor data, such as the design of classifiers for specific error types or the adaptation of statistical-based machine translation methods [51,52,53]. Notable text editing models, including LaserTagger [54], EditNTS [55], PIE [56], and Felix [57], have been applied to sensor-based image captioning tasks, demonstrating promising results in improving the quality of captions generated from sensor data. The process of modifying image captions is a natural extension of applying text editing techniques to sensor-derived image content, including implicit image caption editing [58] and explicit image caption editing [10], which are effective in generating real captions that describe the content of sensor-derived images based on a reference caption.

## 3. ICICD Dataset

In this study, we propose a new inference chain dataset, ICICD, Image Caption Inference Chain Dataset. This dataset is designed to facilitate and enhance image comprehension and natural language processing through the correlation between images, textual descriptions, and critical information capabilities. Raw data from a publicly available dataset, the Flickr30K-EE dataset [10], were utilized for this purpose. A total of 3087 data items from the Flickr30K-EE training set were selected for analysis. The reason for choosing a specific number of data items is that the original dataset is quite large, and we aim to experiment with a small portion of the total data volume, approximately 3%. The data items include image IDs and associated text descriptions. While there are duplicates in the image ID field, the content of the associated text descriptions differs for each data item. The extracted data items involve 2365 different images, providing a rich visual basis for the subsequent generation of object relationship data. The two parts of the ICICD dataset, namely the reference caption and the ground-truth caption, are derived from the original Flickr30K-EE dataset. The object relationship caption is generated by us using a detailed prompt to guide the large multimodal model. The keywords are nouns and verbs extracted from the object relationship caption. The following section provides a more detailed description of the ICICD dataset.

### 3.1. Components of The ICICD Dataset

The inference chain dataset comprises four principal components: the reference caption, object relationship description, keywords, and ground-truth caption. The reference caption and ground-truth caption are abbreviated as Ref-Cap and GT-Cap, respectively. The ECE dataset indicates that there are four principal criteria of association between Ref-Cap and GT-Cap: human-annotated captions, image–caption similarity, caption similarity, and caption differences [10]. Both are written by humans. The scenes described by Ref-Cap are similar to the scene in the image. Ref-Cap and GT-Cap have some degree of overlap and similar caption structures. The differences between Ref-Cap and GT-Cap are more than just one or a few words. The object relationship descriptions are derived from the object relationship data generated using the prompt-guided Qwen-VL model [12]. This constitutes the core of the inference chain, ensuring that the relative positions and dynamic interactions of objects in the image can be accurately captured and described. Keyword extraction is the process of extracting key nouns and verbs from the object relationship descriptions. These words serve as the primary means of comprehending and reconstructing the content of the image, as they encompass the most pivotal objects and actions described. The four components analyze the image in a systematic manner, progressing from a superficial to a profound level of analysis, reasoning in detail and interacting with each other, ultimately forming a comprehensive chain of reasoning.

### 3.2. Create Object Relationship Data

For each image, a detailed English prompt is used to instruct the large multimodal model Qwen-VL to generate a detailed list of spatial relationships and dynamic behaviors between objects in the image. The content of the prompt requires the model to list in detail the relationships and dynamic interactions between all the objects displayed in the image. In addition, the model is required to describe not only simple spatial relationships between objects, such as “object next to object” or “object above object”, but also more complex relationships, such as “object in contact with object”, “object in contact with surface”, or “object in contact with environment”. The model is required to not only describe the simple spatial relationships between objects, such as “object next to object” and “object above object” but also record the actions of any person, animal, or environmental changes. In particular, the prompt requires the model to create a clear and unique description of the relationships and actions between each pair of objects or entities shown in the image.

**Figure 2:**
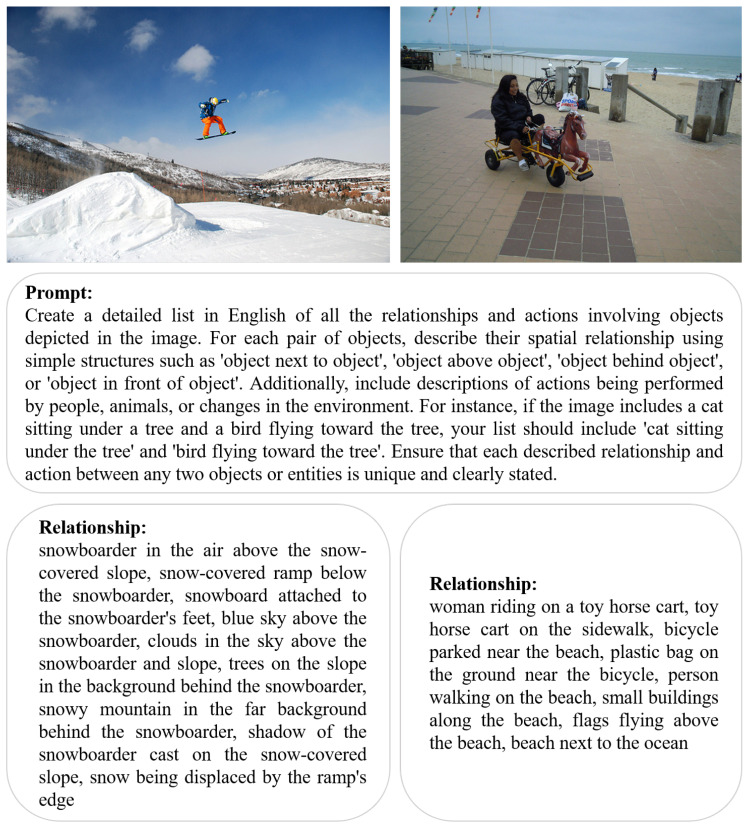
The image and text above show the prompt we designed and the examples of Qwen-VL generating object relationship data based on the prompt guidance and image.

The following two images are presented to illustrate the generation of object-relational data. As shown in the Figure 2, the first image depicts the dynamic activity of a snowboarder on a snow field. The descriptions generated by the Qwen-VL model encompass not only the spatial relationship between the snowboarder and objects such as the snow field and the ski jump, as exemplified by “snowboarder in the air above the snow-covered slope” and “snow-covered ramp below the snowboarder”, but also describe the skier’s movements, such as “snowboard attached to the snowboarder’s feet”, as well as the environment and environmental changes, including “trees on the slope”, “snowy mountain in the far background”, and “snow being displaced by the ramp’s feet”. In addition to describing the spatial relationships between the snowboarder and the surrounding objects, such as the snow-covered ramp below the snowboarder and the trees on the slope, the model also provides insights into the skier’s movements, including the attachment of the snowboard to the skier’s feet. Furthermore, it captures the environmental context, including the presence of a snowy mountain in the background and the displacement of snow by the ramp’s feet. The descriptions encompass not only the relative positions between objects but also the interaction between the activity and the environment, thus helping to complete the depth and spatial layout of the image scene. The second image depicts a scene of a woman in a toy wagon. In this scene, the model details the action “woman riding on a toy horse cart” and indicates the location of the toy horse cart “toy horse cart on the sidewalk”. Furthermore, the model also captures other actions and object relationships, such as “bicycle parked near the beach” and “person walking on the beach”, which contribute to the dynamic elements and background information of the scene.

## 4. Method

### 4.1. Background

The TinyLLaVA framework [11] is designed for small-scale large multimodal models (LMMs) and consists of three main components: a small-scale LLM $F_{\theta }$, a vision encoder $V_{\varphi }$, and a connector $P_{\phi }$. These components work together to process and integrate image and text data, thereby enhancing the model’s performance on various multimodal tasks.

**Small-scale LLM ($F_{\theta }$):** The small-scale LLM takes as input a sequence of text vectors ${\left\{h_{i}\right\}}_{i=0}^{N-1}$ of length *N* in the *d*-dimensional embedding space and outputs the corresponding next predictions ${\left\{h_{i}\right\}}_{i=1}^{N}$. This model typically includes a tokenizer and embedding module that maps input text sequences ${\left\{y_{i}\right\}}_{i=0}^{N-1}$ to the embedding space and converts the embedding space back to text sequences ${\left\{y_{i}\right\}}_{i=1}^{N}$.

**Vision Encoder ($V_{\varphi }$):** The vision encoder processes an input image *X* and outputs a sequence of visual patch features $V={\left\{v_{j}\in R^{d_{x}}\right\}}_{j=1}^{M}$, where $V=V_{\varphi }\left(X\right)$. This encoder can be a Vision Transformer or a Convolutional Neural Network (CNN) that outputs grid features which are then reshaped into patch features.

**Connector ($P_{\phi }$):** The connector maps the visual patch features ${\left\{v_{j}\right\}}_{j=1}^{M}$ to the text embedding space ${\left\{h_{j}\right\}}_{j=1}^{M}$, where $h_{j}=P_{\phi }\left(v_{j}\right)$. The design of the connector is crucial for effectively leveraging the capabilities of both the pre-trained LLM and vision encoder.

The training of TinyLLaVA involves two main stages: pre-training and supervised fine-tuning.

**Pre-training:** This stage aims to align the vision and text information in the embedding space using an image caption format $\left(X,Y_{a}\right)$, derived from multi-turn conversations. Given a target response $Y_{a}={\left\{y_{i}\right\}}_{i=1}^{N_{a}}$ with length $N_{a}$, the probability of generating $Y_{a}$ conditioned on the image is computed as follows:

(1) $$p\left(Y_{a}|X\right)=\prod_{i=1}^{N_{a}}F_{\theta }\left(y_{i}|P_{\phi }\circ V_{\varphi }\left(X\right)\right)$$

The objective is to maximize the log-likelihood autoregressively:

(2) $$\max_{\phi ,\theta^{\prime },\varphi^{\prime }}\sum_{i=1}^{N_{a}}\log F_{\theta }\left(y_{i}|P_{\phi }\circ V_{\varphi }\left(X\right)\right)$$

where $\theta^{\prime }$ and $\varphi^{\prime }$ are subsets of the parameters θ and φ, respectively. This stage allows for the adjustment of partially learnable parameters of both the LLM and vision encoder to better align vision and text information.

**Supervised Fine-tuning:** Using image–text pairs (X,Y) in a multi-turn conversation format $Y=(Y_{q}^{1},Y_{a}^{1},\ldots ,Y_{q}^{T},Y_{a}^{T})$, where $Y_{q}^{t}$ is the human instruction and $Y_{a}^{t}$ is the corresponding assistant’s response, the model maximizes the log-likelihood of the assistant’s responses autoregressively:

(3) $$\max_{\phi ,\theta^{\prime },\varphi^{\prime }}\sum_{i=1}^{N}I\left(y_{i}\in A\right)\log F_{\theta }\left(y_{i}|P_{\phi }\circ V_{\varphi }\left(X\right)\right)$$

where *N* is the length of the text sequence *Y* and $I(y_{i}\in A)=1$ if $y_{i}\in A$ and 0 otherwise. This stage also permits the adjustment of partially learnable parameters of the LLM and vision encoder.

### 4.2. Fine-Tuning of The ECRMM Model

The fine-tuning process for the Explicit Image Caption Reasoning Multimodal Model (ECRMM) involves multiple stages to ensure the model’s effectiveness and efficiency. The optimization process for the ECRMM begins by investigating the potential for reducing memory usage. Two RTX 4090D GPUs (Nvidia, Lanzhou, GS, China) are configured, and the TinyLLaVA-1.5B version is selected as the base model for fine-tuning.

**Figure 3:**
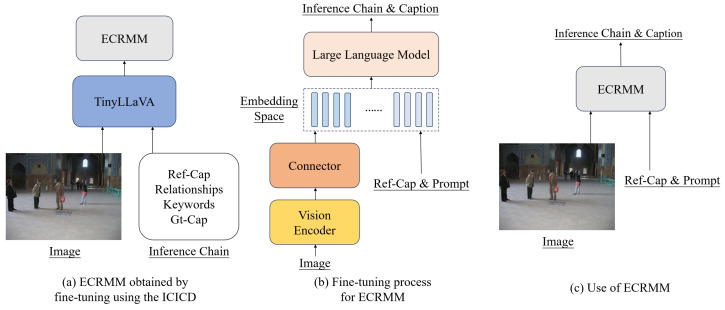
(**a**) represents the ECRMM obtained by fine-tuning TinyLLaVA using the ICICD dataset, (**b**) represents the fine-tuning process of the ECRMM model and the internal structure of the model involved, and (**c**) represents the use of ECRMM to generate the inference chain and caption based on the image and reference.

Figure 3 illustrates the fine-tuning process and the structure of the ECRMM model. As shown in Figure 3a, the ICICD dataset is utilized to fine-tune the TinyLLaVA-1.5B model, resulting in the ECRMM model. During the model fine-tuning process, adjustments to the batch size and epoch are crucial to ensure that the memory footprint does not exceed the total GPU memory. Concurrently, the loss value is monitored closely to ensure that it remains within a reasonable range, thereby optimizing the performance of the ECRMM model. After numerous tests, it was found that the ECRMM model performs best when the loss value is stabilized at approximately 1.2.

Figure 3b depicts the internal structure of the ECRMM model, highlighting the integration of the vision encoder, connector, and LLM. The vision encoder processes the input images, generating visual patch features. These features are then mapped to the text embedding space by the connector, which facilitates the LLM’s ability to generate accurate and detailed captions.

Figure 3c illustrates the use of the ECRMM model to generate inference chains and captions. The model takes an image and reference captions as input, analyzes the object relationships, and extracts keywords to generate a comprehensive and semantically accurate ground-truth caption.

### 4.3. The Method of Inference Chain

First, the entire inference process is based on Ref-Caps, which are descriptions structured to reflect the fundamental scene of the image, thus ensuring high relevance and semantic similarity to the image content. Second, the model generates exhaustive object relationship data based on the images, describing the spatial relationships and dynamic interactions between objects in the images. Subsequently, the model meticulously extracts keywords, mainly nouns and verbs, from the object relationship descriptions. These keywords are crucial for generating the final GT-Cap. The generation of the GT-Cap is the final step of the inference chain. It is not only based on the semantic structure of the images and reference descriptions but also incorporates key action and object information distilled from the object relationships. This generates a content-rich and semantically accurate image summary.

**Figure 4:**
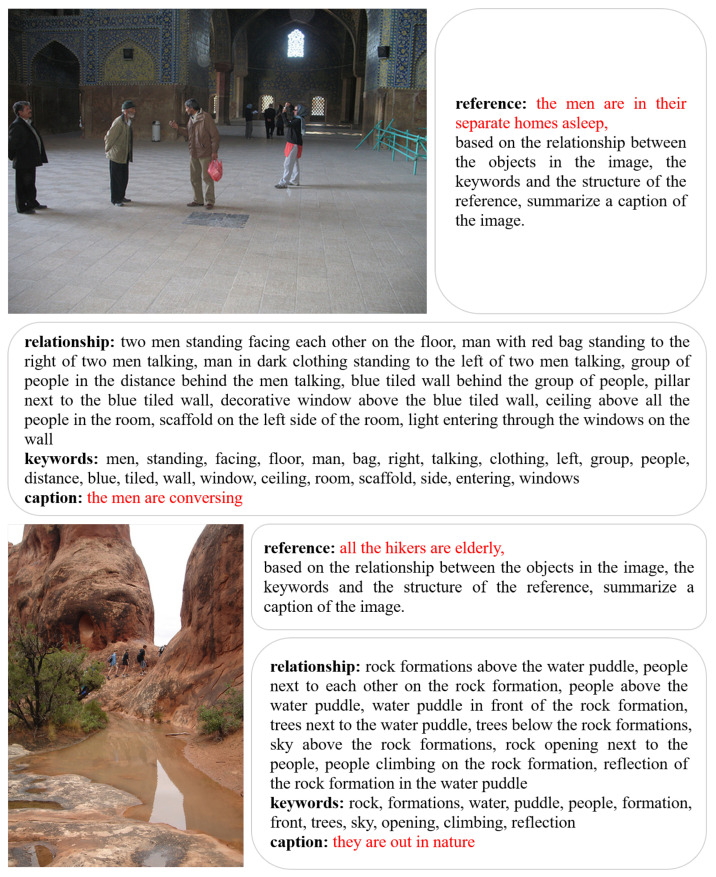
Two examples of the inference chain dataset ICICD are shown here. The inference chain data consists of four main components: the reference caption, the object relationship description, the keywords, and the ground-truth caption. The red text highlights relevant examples of Ref-Cap and GT-Cap.

In order to gain a deeper understanding of the utility of the inference chaining approach, we present two concrete application examples. As shown in Figure 4, the first image depicts several individuals engaged in conversation, with the accompanying caption indicating that the men are in their respective homes asleep. The model analyzes the image and generates a description of the object relationships, including the spatial location of multiple individuals and the background environment. This description may include elements such as “two men standing facing each other on the floor”. The keywords included “men, standing, facing, talking”, which directly affected the generation of the ground-truth caption. The final ground-truth caption was succinct: “the men are conversing”. The second image depicted a number of elderly people in a natural environment; the reference caption was “all the hikers are elderly”. The object relationship descriptions provide detailed information about the relationship between the rocks, puddles, and their surroundings. For instance, the description “rock formations above the water puddle” and “people climbing on the rock formation” provide insight into the spatial arrangement of the elements in the image. The keywords “rock, water, puddle, people, climbing” were instrumental in developing an accurate description of the image. The final ground-truth caption, “they are out in nature”, effectively conveys the theme and activity of the image.

## 5. Experiments

### 5.1. Dataset

In the fine-tuning phase of the ECRMM model, we employ the self-constructed ICICD dataset, a dataset designed for the inference chaining task and comprising a total of 3087 data items. This dataset is created with the intention of providing sufficient scenarios and examples to enable the model to effectively learn and adapt to inference chain processing. In the testing phase of the ECRMM model, we employ the test dataset portion of the publicly available Flickr30K-EE dataset, which contains a total of 4910 data items. This test dataset serves as a standardized benchmark for evaluating the ECRMM model. With this setup, we are able to accurately assess the performance and reliability of the ECRMM model in real-world application scenarios.

### 5.2. Fine-Tuning Details

A total of 2 RTX 4090D GPUs (Nvidia, Lanzhou, GS, China) are employed for the experiments, and the entire fine-tuning process is completed in less than 2 h. During the fine-tuning period, the batch size of the entire model is set to 5 per GPU. Given that we use 2 times gradient accumulation and 2 GPUs, this equates to a global batch size of 20. The model is fine-tuned over 3 training cycles using a cosine annealing scheduler to optimize the decay path of the learning rate. The initial learning rate is set to 2 ×$10^{-5}$ with a weight decay setting of 0, which facilitates the fine-tuning of the model while maintaining the original weight structure. Additionally, a warm-up ratio of 0.03 is employed, whereby the learning rate is gradually increased to a set maximum value at the commencement of training and subsequently decayed according to a cosine curve. In consideration of the storage limitations and efficiency, the model is configured to save every 30 steps and retain only the most recent 3 checkpoints. This approach ensures that the storage space is not overburdened while capturing the crucial progress during the training process.

### 5.3. Evaluation Setup

A test set of the Flickr30K-EE dataset is employed to evaluate the efficacy of our ECRMM. Caption quality assessment is conducted in accordance with existing caption generation efforts, utilizing four generalized evaluation metrics: BLEU-n (1-4) [59], ROUGE-L [60], CIDEr [61], and SPICE [62]. The captions generated are evaluated based on their unique ground-truth CAPTION to assess the quality of the generated captions. Additionally, the METEOR [63] metric is employed to compute the results of our model.

In order to establish a baseline for evaluation purposes, we conduct a comparison between our self-developed ECRMM model and the current state-of-the-art image caption editing models. These include three implicit caption editing models, UpDn-E [19], MN [58], and ETN [9], which are all based on the widely-used UpDn [19] architecture. In addition, we consider four explicit caption editing models. The evaluation baseline comprises five editing models: V-EditNTS, V-LaserTagger, V-Felix, and TIger [10]. V-EditNTS, V-LaserTagger, and V-Felix are obtained by extending three explicit text editing models—EditNTS [55], LaserTagger [54], and Felix [57]—to the ECE framework.

### 5.4. Comparisons with State-of-the-Arts

A comparison was conducted between the ECRMM model and several existing models. As demonstrated in the accompanying Table 1, the ECRMM model exhibits a notable degree of superiority in several assessment metrics. In particular, our model achieved the highest scores on each of the BLEU-1 to BLEU-4, ROUGE-L, CIDEr, and SPICE metrics. The CIDEr score improved from 148.3 to 152.6 for the TIger model, and the SPICE score improved from 32.0 to 32.7. The presented data clearly demonstrate the efficacy of our approach in enhancing semantic comprehension and generating captions that are more closely aligned with the ground-truth caption. The exceptional performance of our model is attributable to its capacity to perform deep semantic parsing and meaningful inference through inference chaining, which is of particular significance when confronted with complex image description tasks.

Ref. [10] describes state-of-the-art TIger models and provides a comprehensive evaluation of different models for related tasks using a variety of metrics. Our study cites data from [10] and uses the same evaluation criteria and benchmarks to evaluate the ECRMM model. When our ECRMM model is compared to these state-of-the-art models, including TIger, our ECRMM model demonstrates superior performance on all metrics. The purpose of this comparison is twofold: first, to list the current state-of-the-art models and their performance for the task in question; and second, to demonstrate the superior performance of the ECRMM model for the task in question compared to the current state-of-the-art models.

In addition to the traditional evaluation metrics, we introduce the METEOR score to further validate the performance of the model. This metric demonstrates that our model also performs well, with a METEOR score of 19.5. These results not only provide a new benchmark for future research but also provide strong technical support for understanding and generating high-quality image descriptions.

### 5.5. Ablation Study

In our ablation study, we compare five methods: w/o all, w/o inference chain, w/o relationship, w/o keywords, and ECRMM with a complete inference chain. The abbreviations for w/o inference chain, w/o relationship, and w/o keywords are w/o i, w/o r, and w/o k, respectively.

As shown in Table 2, from Bleu-1 to Bleu-4, it is evident that all variants of the model have demonstrated an improvement compared to w/o all, particularly in the model ECRMM, which incorporates a comprehensive inference chain. Bleu-4 has reached 15.8, which is higher than w/o all’s 14.9. Additionally, the three metrics of ROUGE-L, CIDEr, and SPICE, with the exception of w/o i, which lacks an inference chain, have also exhibited an upward trend. All other models have also demonstrated improvement compared to w/o all. This indicates that both object-relational descriptions and keywords in the inference chain have a positive impact on model performance. This emphasizes the important role of object-relational sentences and keywords in improving the semantic accuracy, syntactic correctness, and overall quality of the model-generated image descriptions.

The complete inference chain provides the model with richer contextual information, which leads to optimal performance on all evaluation metrics. While w/o i scores lower than w/o all on some metrics, it also scores higher than w/o all on others. This demonstrates that even in the absence of additional inputs of semantic information, the large multimodal model itself is powerful enough to achieve its diverse data generalization ability. The model remains adaptive and sensitive to the characteristics of different datasets without the aid of additional information. However, the absence of sufficient contextual information results in the model underperforming the original model on metrics such as CIDEr and SPICE, which are more focused on evaluating the uniqueness of the description and the comprehensiveness of the information.

Furthermore, it can be demonstrated that the keyword-only w/o r does not outperform the object relationship description—only w/o k in all metrics. This phenomenon suggests that the object relationship sentence provides spatial and interactional relationships between objects in an image, which is a key component for understanding the content of an image. This description has a more direct impact on the semantic structure of the generated semantics, as it provides a comprehensive semantic parsing of the image scene, enabling the model to more accurately comprehend the dynamic relationships and layout in the image. While keywords are capable of highlighting the primary elements and actions in an image, they provide more one-sided information or are limited to specific objects and actions than object relationship clauses, missing the interactions and spatial relationships between objects. This underscores the significance of spatial and dynamic relationships between objects in comparison to keyword annotation alone in image description tasks.

### 5.6. Sensitivity Analysis of Data Volume

**Figure 5:**
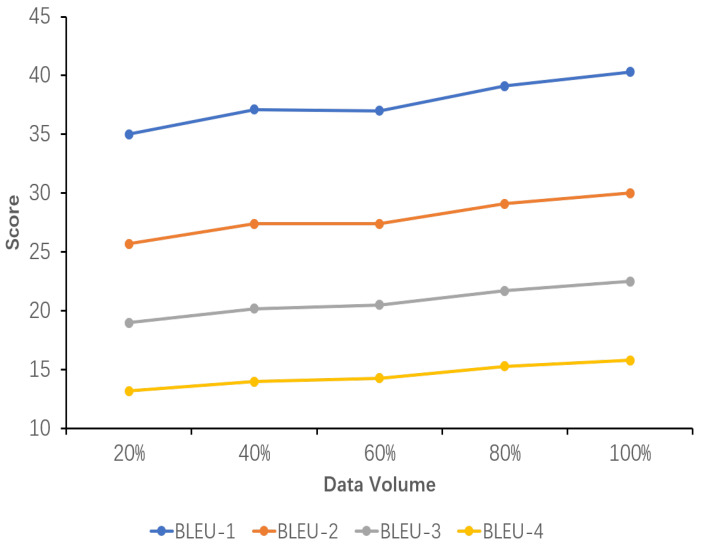
Sensitivity analysis of the performance of the fine-tuned model using datasets with different data volumes. Percentages represent the percentage of the ICICD dataset accounted for. The variation of the BLEU-n(1-4) scores with increasing data volume is shown here.

**Figure 6:**
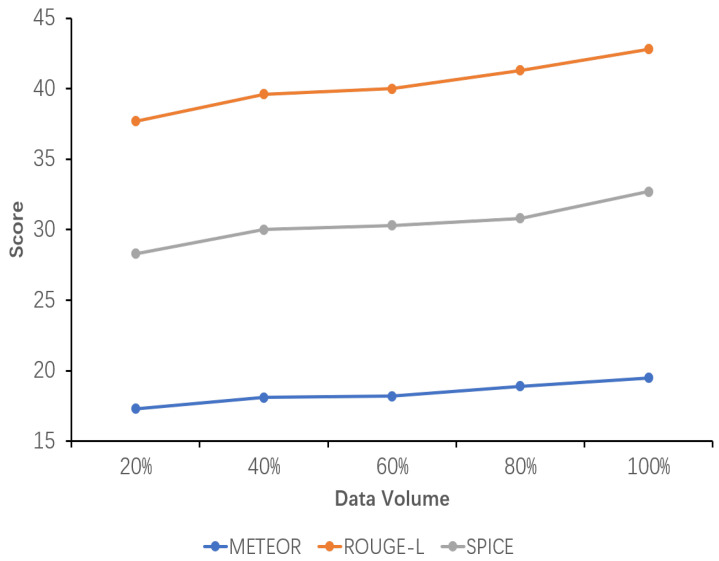
Sensitivity analysis of the performance of the fine-tuned model using datasets with different data volumes. The variation in the METEOR, ROUGE-L, and SPICE scores with increasing data volume is shown here.

**Figure 7:**
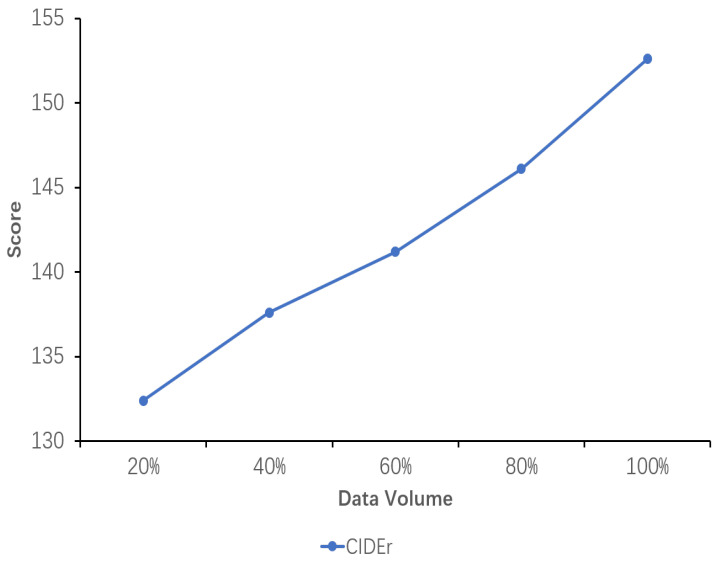
Sensitivity analysis of the performance of the fine-tuned model using datasets with different data volumes. The variation in the CIDEr scores with increasing data volume is shown here.

The results of the sensitivity analysis of data volume demonstrate the impact of data volume on the performance of the explicit image caption Reasoning task, where the dataset used has a complete inference chain. Five proportions of data volume were set, namely 20%, 40%, 60%, 80%, and 100%. As can be seen from the images in Figure 5, Figure 6 and Figure 7, all the evaluation metrics show significant improvement as the amount of training data increases. This indicates that on smaller datasets, the model is prone to learning noise and chance laws in the data rather than universally applicable laws, with the risk of overfitting. With the addition of more data, the model is able to learn more diverse image features and linguistic expressions, improving its ability to generalize to different scenes, objects, and actions. Larger datasets provide richer contexts and examples, enabling the model to better capture and learn the nuances in image descriptions.

Concurrently, it is observed that while the model’s performance exhibits a gradual improvement from 20% to 80% of the data volume, it is not until 100% of the data volume is utilized that the performance exceeds the original model’s TIger score. This amount of data is sufficiently limited in comparison to the original Flickr30K-EE dataset yet sufficiently extensive to enhance the model’s performance on this ECR task. This outcome validates the appropriateness of the selected data amount setting.

### 5.7. Sensitivity Analysis of the Number of Object Relationship Sentences

**Figure 8:**
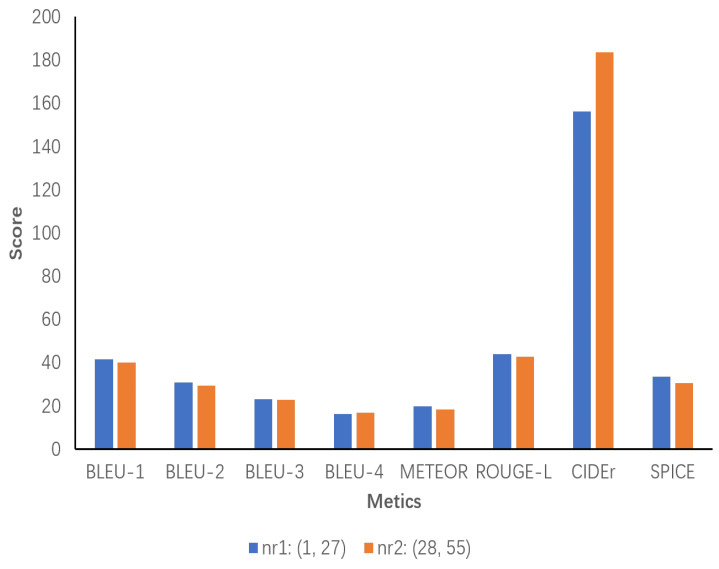
Sensitivity analysis of the number of object relation sentences generated by the ECRMM model on the test set. nr1 ranges from 1 to 27 and nr2 ranges from 28 to 55.

A sensitivity analysis is conducted on the data generated by the model ECRMM on the test set to ascertain the impact of the number of object-relative sentences on the performance of the evaluation metrics. First, the number of object-relative sentences is counted and divided into two ranges of phrase count values, designated as nr1 and nr2, based on the minimum and maximum values. These values are then evaluated separately for each range. According to the statistical analysis, the minimum and maximum values for the number of object-relative sentences are 1 and 55, respectively. In Figure 8, the results demonstrate that nr1 exhibits high performance, with all metrics outperforming the performance of the model ECRMM. This indicates that a moderate number of object-relative sentences can effectively support the generation of high-quality image descriptions. While Nr2 still performs well on CIDEr (183.6), it is evident that the descriptions are less unique and relevant than those generated by Nr1. This is evidenced by the decrease in Bleu and METEOR scores, with SPICE decreasing to 30.6. This suggests that the semantic accuracy of the descriptions has decreased.

**Figure 9:**
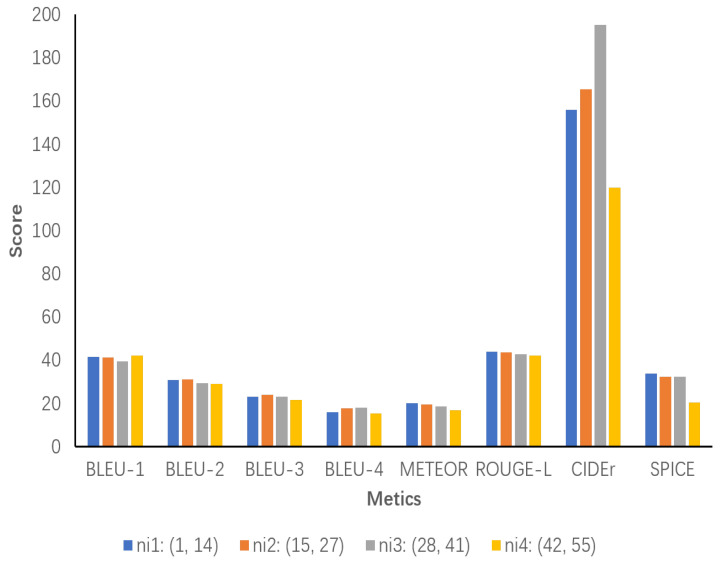
All of the object relationship sentence numbers are grouped into smaller intervals for more precise analysis. ni1 ranges from 1 to 14, ni2 ranges from 15 to 27, ni3 ranges from 28 to 41, and ni4 ranges from 42 to 55.

We then divide nr1 into two intervals, ni1 and ni2, and nr2 into ni3 and ni4 and then again score each of the four intervals. As shown in Figure 9, the results indicate that ni1 performs better in most of the metrics, and ni2 shows a significant increase in Bleu-4 and CIDEr compared to interval 1. This suggests that increasing the number of object relationship clauses within the interval helps the model to generate more accurate and information-rich descriptions. However, ni3 shows a slight decrease in Bleu-1, METEOR, and SPICE, although the CIDEr metric is high at 195.3, indicating that the descriptions become complex or overly detailed due to the excessive number of object relationship sentences. Ni4 exhibits a significant decrease in performance, indicating that excessive object relationship sentences cause the descriptions to be redundant or incorrectly generated. Redundancy or generation errors in the description affect the coherence and accuracy of the description.

The results of our data analysis indicate that the optimal interval for object-relational sentences is between ni1 and ni2, which encompasses approximately 15 sentences. Additionally, a high concentration of sentences converging towards ni4 is detrimental to performance, particularly in terms of coherence and semantic accuracy. The number of object-relational sentences has a direct impact on the quality of the generated image descriptions. An excess of object-relational descriptions can lead to negative effects, whereas an appropriate number of object-relational sentences can improve the richness and accuracy of the descriptions.

### 5.8. Sensitivity Analysis of Inference Chain Length

**Figure 10:**
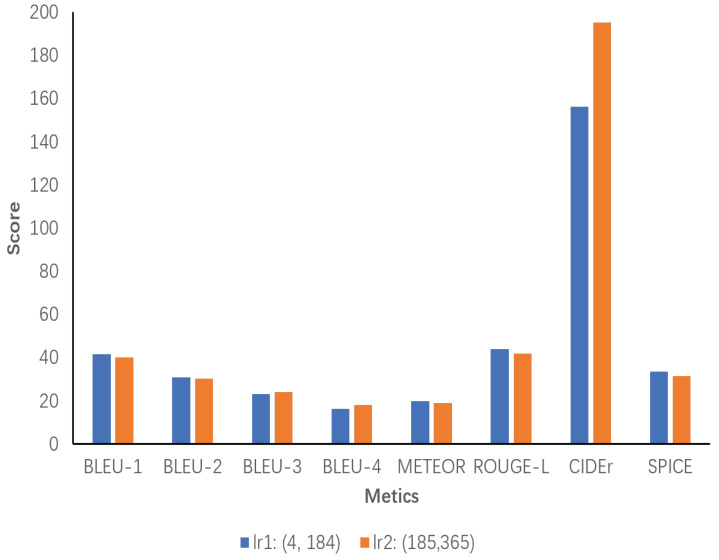
Sensitivity analysis of the length of inference chains generated by the ECRMM model on the test set. lr1 ranges from 4 to 184, and lr2 ranges from 185 to 365.

Additionally, a sensitivity analysis of inference chain length is conducted. The number of words in all inference chains generated by the model ECRMM on the test set is initially counted and then divided into two ranges based on the minimum and maximum values, range1 and range2. Each range is evaluated separately, with the results presented separately. According to our statistical analysis, the minimum and maximum values of inference chain lengths are 4 and 365, respectively. In Figure 10, lr1 demonstrates slight superiority over lr2 in most metrics, particularly Bleu-1, ROUGE-L, and SPICE, which indicates that shorter inference chains are sufficient for high-quality descriptions within this range. It can be concluded that chains of a sufficient length are sufficient to provide high-quality descriptions in this range. Despite its excellent performance on CIDEr, lr2 underperforms compared to lr1 on most metrics. This is due to the fact that excessively long inference chains increase the complexity of the descriptions without necessarily improving their quality.

**Figure 11:**
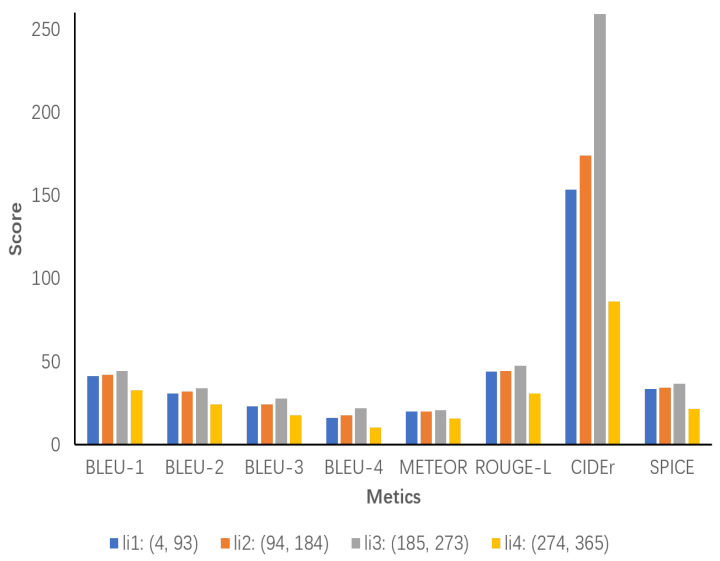
All inference chain lengths are divided into smaller intervals for more exact analysis. li1 ranges from 4 to 93, li2 ranges from 94 to 184, li3 ranges from 185 to 273, and li4 ranges from 274 to 365.

In Figure 11, the results indicate that both li1 and li2 demonstrate a gradual increase in performance, particularly with regard to CIDEr and SPICE. This suggests that a moderately increasing inference chain length may be beneficial in improving the information richness and semantic accuracy of the descriptions. In comparison, li3 is the best performer, reaching a peak on almost all metrics. Notably, it also scores extremely high on CIDEr. Furthermore, the SPICE metric indicates that the inference chain length in this interval is optimal and able to provide sufficient detail and complexity to generate high-quality image descriptions. However, the performance of li4 is significantly degraded, which is likely due to the redundancy of descriptions or semantic clutter caused by excessively long inference chains, thus affecting all performance metrics.

The length of the inference chain has a significant impact on the quality of image descriptions generated by the model. An inference chain that is too short does not provide sufficient information, while an inference chain that is too long results in redundant or degraded descriptions. Inference chains with a length of approximately 230 words around the optimal length demonstrate superior performance in almost all metrics, providing detailed and accurate descriptions. In contrast, inference chains with a length of approximately 300 words demonstrate degraded performance.

### 5.9. Analysis of Keyword Generation

To further evaluate the performance of the model, we assess the degree of match between the keywords generated by the ECRMM model and the keywords generated by the Qwen-VL model by analyzing the precision, recall, and F1 score. For this purpose, we randomly select 100 items from the data generated by the ECRMM model in the test set and use the Qwen-VL model to generate keywords for these 100 images. Given the exemplary performance of the Qwen-VL model across all metrics, we utilize the keywords generated by the Qwen-VL model as the benchmark for comparison. Using the keywords from the Qwen-VL model as a standard reference, we perform the calculation for each sample to determine how well the ECRMM model performs in generating accurate and comprehensive keywords. As shown in Table 3, the ECRMM model achieves the highest precision, recall, and F1 score for keyword matching, with values of 0.89, 0.73, and 0.80, respectively. This indicates that the model is highly accurate in identifying the correct keywords, particularly in image description tasks, and has a superior ability to capture the most relevant keywords.

However, the average precision of keyword matching is 0.49, while the average recall is 0.55. This indicates that the model performs well in both generating keywords that are indeed relevant and covering all relevant keywords. However, it does not reach very good performance. The average F1 score is 0.49, which indicates that there is still room for improvement in the overall effectiveness of the model. This also reflects the model’s tendency towards volatility in performance. However, under certain conditions, the model has the potential to achieve excellent results.

### 5.10. Comparison of Qualitative Results

**Figure 12:**
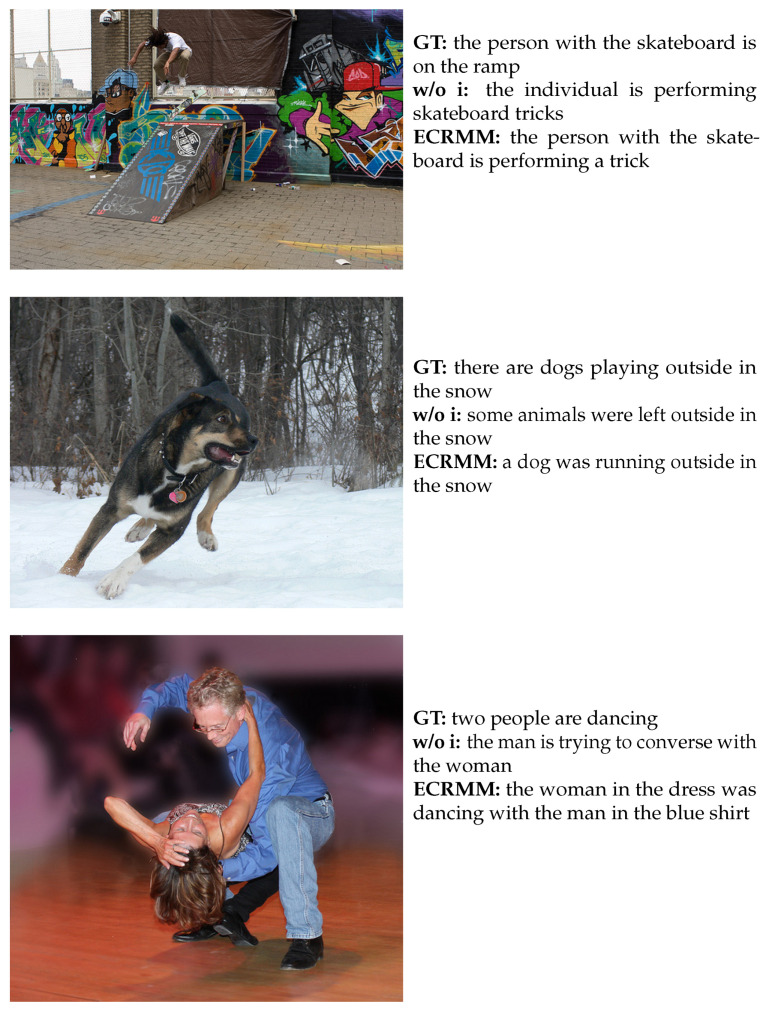
Examples of captions generated by our ECR approach and the original method, as well as the corresponding ground-truths.

The model’s performance in terms of qualitative results is noteworthy. As shown in Figure 12, the descriptions generated by ECRMM are more consistent with GT’s utterance structure, as evidenced by the descriptions generated by ECRMM in the first and second images. ECRMM employs inference chaining to enhance the accuracy of the descriptions generated, as exemplified by the increased precision of “a dog” in the second image compared to “some animals” and the enhanced accuracy of “dancing” in the third image compared to “trying to converse”. In the second image, the description “a dog” is more accurate than “some animals”. Similarly, in the third image, “dancing” is more accurate than “trying to converse”. For instance, “a dog” in the second image is more accurate than “some animals”, and “dancing” in the third image is more accurate than “trying to converse”. The ECRMM model is capable of generating more descriptive and detailed text through the use of inference chains. In the second image, the “was running outside” generated by ECRMM is more focused on the primary action depicted in the image than the “were left outside” generated by w/o i. The “in the dress” and “in the blue shirt” generated in the third image capture additional details in the image and provide a highly specific description.

In comparison to the w/o i model, the ECRMM model exhibits notable enhancements in terms of detail and accuracy. This is due to its capacity to discern and delineate multiple objects and their interrelationships within an image. Furthermore, in contrast to GT, ECRMM provides more comprehensive information in certain instances, thereby exemplifying the model’s aptitude to comprehend and describe the dynamics of a scene.

## 6. Conclusions

This paper introduces the Explicit Image Caption Reasoning method and discusses its application and advantages in specific image captioning tasks, such as the ECE task. It presents the ICICD dataset based on this approach and uses it to fine-tune the large multimodal model TinyLLaVA to obtain the ECRMM model. The ECRMM model is subjected to extensive analysis and ablation experiments, and the results demonstrate a significant improvement in its performance. The method produces more detailed and higher-quality captions by understanding the content of the image in greater depth. Our study not only validates the effectiveness of the Explicit Image Caption Reasoning method in image caption generation but also opens up new avenues for future research, especially in accurately processing visual content.

## Figures and Tables

**Table 1:** Performance of our model and other models on Flickr30K-EE. “Ref-Caps” denotes the quality of given reference captions. In order to facilitate the comparisons of future models with our method, the ECRMM evaluate a METEOR score of 19.5 on the Flickr30K-EE test set.

Method	BLEU-1	BLEU-2	BLEU-3	BLEU-4	ROUGE-L	CIDEr	SPICE
Ref-Cap	34.7	24.0	16.8	10.9	36.9	91.3	23.4
UpDn [10,19]	25.6	16.1	10.4	6.3	30.1	71.0	21.4
UpDn-E [10,19]	33.9	24.7	18.3	12.5	41.1	129.1	29.8
MN [10,58]	30.0	20.0	13.6	8.6	34.9	91.1	25.2
ETN [9,10]	34.8	25.9	19.6	13.7	41.8	143.3	31.3
V-EditNTS [10,55]	38.0	27.0	20.1	13.8	40.2	129.1	28.7
V-Felix [10,57]	21.1	16.7	13.5	10.1	38.0	127.4	27.8
V-LaserTagger [10,54]	30.8	20.8	15.0	10.5	34.9	104.0	27.3
TIger [10]	38.3	28.1	21.1	14.9	42.7	148.3	32.0
ECRMM	**40.3**	**30.0**	**22.5**	**15.8**	**42.8**	**152.6**	**32.7**

**Table 2:** Ablation experiments and comparison of different compositions of inference chain. Higher values indicate better results. ”w/o i”, “w/o r”, and “w/o k” are abbreviations for “w/o inference chain”, “w/o relationship”, and “w/o keywords”, respectively.

Method	BLEU-1	BLEU-2	BLEU-3	BLEU-4	METEOR	ROUGE-L	CIDEr	SPICE
w/o all	38.3	28.1	21.1	14.9	-	42.7	148.3	32.0
w/o i	40.2	29.2	21.3	14.9	19.1	41.8	143.5	31.7
w/o r	40.3	29.8	22.1	15.4	19.4	42.7	149.3	32.0
w/o k	40.3	30	22.3	15.7	19.4	42.7	149.3	32.4
ECRMM	40.3	30	22.5	15.8	19.5	42.8	152.6	32.7

**Table 3:** Performance analysis of ECRMM conducted on 100 samples. Precision, recall, and F1 score are calculated for each sample by generating keywords and referring to Qwen-VL generation.

	Precision	Recall	F1 Score
Highest	0.89	0.73	0.80
Average	0.49	0.55	0.49

## Data Availability

Data are contained within the article.
